# Excellent outcome of stem cell transplantation for sickle cell disease

**DOI:** 10.1007/s00277-023-05447-4

**Published:** 2023-09-19

**Authors:** Tanja Vallée, Irene Schmid, Lisa Gloning, Martina Bacova, Jutta Ahrens, Tobias Feuchtinger, Christoph Klein, Vincent D. Gaertner, Michael H. Albert

**Affiliations:** Department of Pediatrics, Dr. Von Hauner Children’s Hospital, University Hospital, LMU Munich, Lindwurmstr. 4, 80337 Munich, Germany

**Keywords:** Sickle cell disease, Stem cell transplantation, Haploidentical, Busulfan

## Abstract

Many sickle cell disease (SCD) patients lack matched family donors (MFD) or matched unrelated donors (MUD), implying haploidentical donors (MMFD) as a logical donor choice. We used a reduced toxicity protocol for all donor types. We included 31 patients (2–22 years) with MFD (*n* = 15), MMFD (10), or MUD (6) HSCT and conditioning with alemtuzumab/ATG, thiotepa, fludarabine and treosulfan, and post-transplant cyclophosphamide for MMFD. After the initial six patients, treosulfan was replaced by targeted busulfan (AUC 65–75 ng*h/ml). After a median follow-up of 26 months (6–123), all patients are alive and off immunosuppression. Two MMFD patients experienced secondary graft failure with recurrence of SCD, both after treosulfan conditioning. Neither acute GVHD ≥ °III nor moderate/severe chronic GVHD was observed. The disease-free, severe GVHD-free survival was 100%, 100%, and 80% in the MFD, MUD, and MMFD groups, respectively (*p* = 0.106). There was a higher rate of virus reactivation in MMFD (100%) and MUD (83%) compared to MFD (40%; *p* = 0.005), but not of viral disease (20% vs 33% vs 13%; *p* = 0.576). Six patients had treosulfan-based conditioning, two of whom experienced graft failure (33%), compared to 0/25 (0%) after busulfan-based conditioning (*p* = 0.032). Donor chimerism was ≥ 80% in 28/31 patients (90%) at last follow-up. Reduced toxicity myeloablative conditioning resulted in excellent overall survival, negligible GVHD, and low toxicity among all donor groups in pediatric and young adult patients with SCD.

**Supplementary Information:**

The online version contains supplementary material available at 10.1007/s00277-023-05447-4.

## Introduction

Sickle cell disease (SCD) has an incidence of approximately 300,000 annual births worldwide and results in reduced life expectancy and impaired quality of life, even with best available supportive care in high-resource countries [1, 2]. Novel disease-modifying drugs have recently become available, but their long-term protective effect on SCD-related organ damage is unclear [3]. Potentially curative gene-modifying treatments are in clinical testing and early efficacy results look promising, but long-term safety still needs to be demonstrated [4, 5]. Even if approved, these treatments will not be available to the majority of SCD patients for socio-economic reasons [6]. Allogeneic hematopoietic stem cell transplantation (HSCT) is currently the only curative option for symptomatic SCD and can improve quality of life of affected patients and families [7, 8], but can be associated with significant therapy-related morbidity and mortality.

HSCT from matched family donors (MFD) is the standard of care for patients with SCD in some high-resource countries like Germany [9], but is more controversially discussed elsewhere [10, 11]. Excellent survival with low rates of graft versus host disease (GVHD) and improved post HSCT quality of life has been demonstrated [8, 12–14]. For patients lacking a MFD, it is often impossible to identify a matched or partially matched unrelated donor (MUD) [15]. Therefore, HSCT from HLA-haploidentical mismatched family donors (MMFD) is a relevant alternative [16]. HSCT from MMFD was increasingly successful over the last years due to improved methods of GVHD prevention by in vitro or in vivo lymphodepletion [17–20]. In vivo manipulation of alloreactive T-cells with post transplantation cyclophosphamide (PTCY) is attractive because of low GVHD rates reported in malignant disorders and its minimal economic impact, making it a feasible option also at HSCT centers in low to middle-income countries [6, 16, 21]. The published evidence of haploidentical HSCT with PTCY in SCD is growing but still limited [22]. Reported series often comprised pediatric as well as adult patients, included diseases other than SCD, or reported high graft-failure rates [19, 21, 23].

We hypothesized that by using a reduced toxicity, myeloablative chemotherapy-based HSCT protocol with intensive immunosuppression determined by donor/recipient HLA mismatch, we could achieve high survival rates and low GVHD incidence in our SCD patients. This manuscript reports the results of this protocol with MFD, MUD, and MMFD in pediatric, adolescent, and young adult SCD patients.

## Methods

### Study design

This was a retrospective, chart-based study. All patients or their respective caregivers gave written informed consent to the treatment according to this institutional protocol and to data storage and analysis via the German Pediatric Registry for Stem cell Transplantation.

### Patients and donors

All pediatric and young adult patients with SCD undergoing allogeneic HSCT at our center from 2012 to 2022 were included with no pre-defined exclusion criteria other than terminal organ damage. The donor choice hierarchy was HLA-match (MFD > 10/10 MUD > 9/10 MUD > MMFD), followed by CMV serostatus, blood type, and donor age. Three patients with MMFD and two with MUD were previously reported [15, 20]. All patients had received ongoing treatment with hydroxycarbamide before HSCT according to national guidelines [9], which was stopped immediately before conditioning, and all patients had three partial exchange transfusions to lower HbS levels to approx. 30–35% in the week before conditioning,

### Procedures

Patients receiving HSCT from MMFD were conditioned with alemtuzumab 2 × 0.2 mg/kg/day (d-9 to d-8), thiotepa 2 × 5 mg/kg (d-8), fludarabine 5 × 30 mg/m^2^ (d-7 to d-3), treosulfan 3 × 14 g/m^2^ (d-7 to d-5), and cyclophosphamide 2 × 14.5 mg/kg/d (d-3 to d-2). After the initial five patients, treosulfan was replaced by targeted intravenous busulfan (weight-based dosing, four times per day; d-7 to d-5) aiming at an AUC of 65–75 ng*h/ml due to institutional concerns about insufficient myeloablation. MUD and MFD recipients were conditioned with thiotepa 2 × 5 mg/kg (d-7), fludarabine 5 × 30 mg/m^2^ (d-6 to d-2), and busulfan with a target AUC of 65–75 ng*h/ml (d-6 to d-4, four times daily, pharmacokinetics after the first dose), except for one MUD recipient who received treosulfan. All MFD and three MUD recipients received serotherapy with ATG Grafalon® (Neovii, Germany) 3 × 15 mg/kg (d-4 to d-2). The three remaining MUD patients received alemtuzumab 4 × 0.2 mg/kg (d-6 to d-3). This was due to a change in institutional policies towards alemtuzumab because of its efficacy and GVHD prevention properties in children with non-malignant diseases [24]. A schematic table of the conditioning regimens is provided as supplemental Table 1. In MMFD transplants, GVHD prophylaxis consisted of post-transplant cyclophosphamide (PTCY) 50 mg/kg/day on d + 3 and d + 4, followed by mycophenolate mofetil (MMF; d + 5 to d + 35) and tacrolimus (d + 5 to d + 100, then tapered). MUD and MFD transplant recipients received immunosuppression with cyclosporine A (d-1 to d + 100, then tapered) and MMF (d0 to d + 28). Bone marrow was the preferred stem cell source and was used in all patients but one MUD recipient. Supportive therapy was performed according to institutional standards including levetiracetam seizure prophylaxis, acyclovir (but not letermovir) antiviral prophylaxis, weekly blood PCR screening for CMV, EBV, and adenovirus, and a platelet transfusion level at 10 × 10^9/l.

### Definitions

Acute GVHD was staged according to modified Glucksberg criteria and chronic GVHD according to NIH consensus standards [25, 26]. Viral screening was performed by weekly blood PCR for CMV, EBV, and adenovirus. Viral reactivation was defined as positive blood PCR without clinical symptoms. Veno-occlusive disease (VOD) was graded according to EBMT criteria [27]. Neutrophil and platelet engraftment were defined as the first of 3 consecutive days with ≥ 0.5 × 10/l neutrophils and the first of 7 consecutive days with ≥ 50 × 10/l platelets in peripheral blood, respectively. Hepatic or renal toxicity was defined as Common Terminology Criteria for Adverse Events (CTCAE) ≥ 3 for alanine aminotransferase or total serum bilirubin, and creatinine, respectively. Event-free survival (EFS) was defined as disease-free (no SCD symptoms) and severe GVHD-free (no. III–IV acute GVHD or moderate/severe chronic GVHD) survival.

### Statistics

Non-parametric data are presented as median and interquartile range (IQR) unless otherwise noted. Survival probabilities were compared using the log-rank (Mantel-Cox) test, categorical variables using Chi-square, and numerical variables using one way ANOVA. *p*-values < 0.05 were considered statistically significant. Statistical analyses were performed using GraphPad Prism 7.0 (San Diego, CA).

## Results

### Patient and transplant characteristics

Overall, 31 patients received a first allogeneic HSCT at a median age of 8 years (range 2–22), 15 from a MFD, six from a MUD, and ten from a MMFD. All had previously experienced severe SCD-related complications except one MFD patient who was only mildly symptomatic. All ten MMFD were parental HLA-haploidentical donors. Four MUD donors were 9/10 HLA-matched (all with an HLA-A antigen mismatch). All grafts (except one MUD) were bone marrow, containing a median of 2.8, 6.2, and 5.1 * 10^8^ total nucleated cells/kg in the MFD, MUD, and MMFD transplants, respectively (*p* < 0.001). Two MMFD recipients had donor-specific anti-HLA antibodies with a mean fluorescence intensity > 5000 and received three to five sessions of plasmapheresis and one dose of rituximab (375 mg/m^2^) pre HSCT in line with EBMT recommendations [28], which resulted in marked decrease of antibody levels to less than 1000. Both received treosulfan conditioning, and one of these patients had very late secondary graft failure at 6 years post HSCT as described below. Detailed patient and transplant characteristics are presented in Table 1.

**Table 1:** Patient and transplant characteristics

	All patients		MFD		MUD		MMFD	
*n* =	31		15		6		10	
Patient characteristics								
Sex, *n* (female/male)	13/18		2/13		3/3		8/2	
Age, years (median, range)	8	(2–22)	8	(2–15)	8	(3–17)	7	(3–22)
< 12 years, *n*	24		12		4		8	
12–18 years	5		3		2		-	
> 18 years	2		-		-		2	
SCD complications, *n* (%)	30/31	(97%)	14/15	(93%)	6/6	(100%)	10/10	(100%)
Vaso-occlusive crises	29/31	(94%)	13/15	(87%)	6/6	(100%)	10/10	(100%)
Stroke	4/31	(13%)	1/15	(7%)	1/6	(17%)	2/10	(20%)
Acute chest syndrome	13/31	(42%)	6/15	(40%)	3/6	(50%)	4/10	(40%)
RBC alloimmunization	3/31	(10%)	-		-		3/10	(30%)
Other^a^	21/31	(68%)	10/15	(67%)	5/6	(83%)	6/10	(60%)
Anti-donor HLA antibodies, *n* (%)	2/31	(6%)	0/15	(0%)	0/6	(0%)	2/10	(20%)
Transplant characteristics								
Donor age, years (range)	26	(1–57)	10	(1–52)	36	(21–48)	35	(26–57)
HLA match, *n* (%)								
10/10			15/15	(100%)	2/6	(33%)	-	
9/10			-		4/6^b^	(67%)	-	
7/10			-		-		2/10	(20%)
5/10			-		-		8/10	(80%)
Female donor/male recipient	8/31	(26%)	5/15	(33%)	3/6	(50%)	0/10	(0%)
Blood type mismatch (none/minor/major)	20/4/7		8/3/4		4/0/2		8/1/1	
CMV status, recipient/donor, *n* (%)								
− / −	2/31	(6%)	2/15	(13%)	-		-	
+ / − , − / + , + / +	29/31	(94%)	13/15	(87%)	6/6	(100%)	10/10	(100%)
Bone marrow as stem cell source, *n* (%)	30/31	(97%)	15/15	(100%)	5/6	(83%)	10/10	(100%)
Graft composition, median (IQR)								
TNC * 10^8/kg	4.4	(2.8–5.2)	2.8	(2.4–4.1)	6.2	(5.1–7.3)	5.1	(4.4–5.6)
CD34 + *10^6/kg	4.2	(3.1–7.0)	3.5	(3.2–5.1)	8.1	(3.7–9.7)	3.9	(3.2–6.2)
CD3 + *10^6/kg	28.4	(19.5–38.7)	20.0	(16.6–28.7)	49.9	(31.3–75.3)	30.3	(26.0–50.3)
Conditioning, *n* (%)								
BU-FLU-TT	20/31	(65%)	15/15	(100%)	5/6	(83%)	-	
BU-FLU-TT-CY	5/31	(16%)	-		-		5/10	(50%)
TREO-FLU-TT	1/31	(3%)	-		1/6	(17%)	-	
TREO-FLU-TT-CY	5/31	(16%)	-		-		5/10	(50%)
Busulfan tAUC, ng*h/ml, median (IQR)	66	(61–67)	66	(63–68)	65	(61–66)	63	(61–67)
Serotherapy, *n* (%)								
Alemtuzumab	13/31	(42%)	-		3/6	(50%)	10/10	(100%)
ATG	18/31	(58%)	15/15	(100%)	3/6	(50%)	-	
GVHD prophylaxis, *n* (%)								
CSA, MMF			15/15	(100%)	6/6	(100%)	-	
PTCY, TAC, MMF			-		-		10/10	(100%)

*ATG* anti-thymocyte globulin, *BU* busulfan, *CSA* cyclosporine A, *CY* cyclophosphamide, *FLU* fludarabine, *IQR* inter quartile range, *MMF* mycophenolate mofetil, *PTCY* post-transplant cyclophosphamide, *RBC* red blood cell, *SCD* sickle cell disease, *TAC* tacrolimus, *tAUC* total area under the curve, *TNC* total nucleated cells, *TREO* treosulfan, *TT* thiotepa

^a^Other complications included: osteomyelitis, arterial hypertension, osteonecrosis, hemolysis, severe infections and psychiatric alterations

^b^All four had an antigen mismatch at HLA-A

### Outcome

**Fig. 1:**
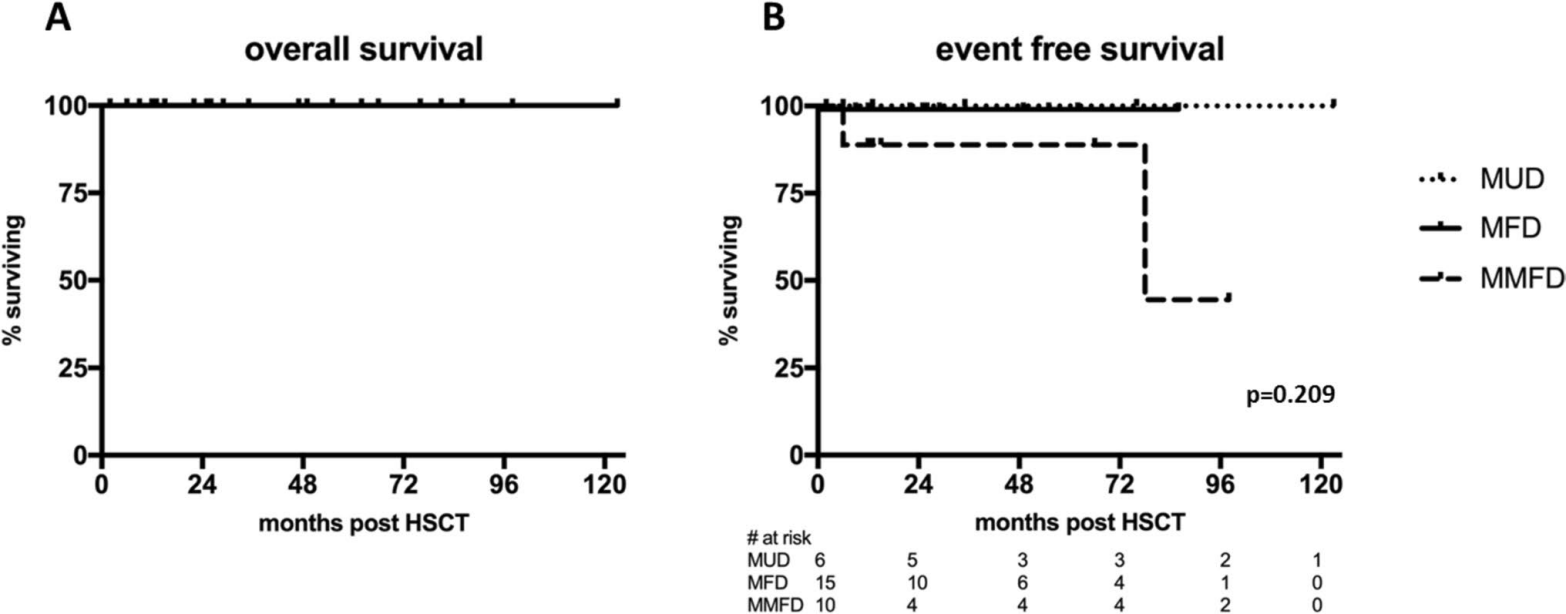
Overall and event-free survival. **A** Overall survival of the entire cohort. **B** Event-free survival (events: III–IV acute GVHD, moderate/severe chronic GVHD, SCD relapse, death) by donor groups

**Fig. 2:**
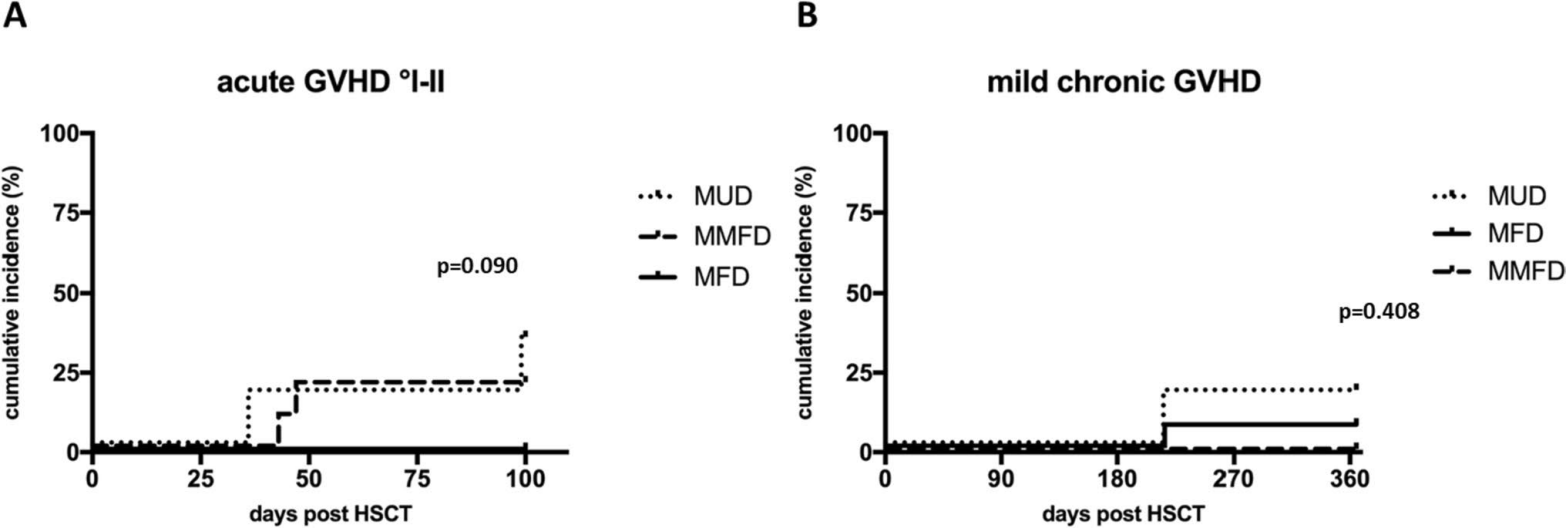
GVHD. Cumulative incidence of (**A)** I–II acute GVHD and (**B)** mild chronic GVHD by donor groups. There was neither acute GVHD °III–IV nor mild/moderate chronic GVHD

After a median follow-up of 26 months (range 6–123), all 31 patients are alive (Fig. 1A) and off immunosuppression. Two patients in the MMFD group experienced secondary graft failure with a relapse of sickle cell disease symptoms; both had treosulfan conditioning. There were two patients each with acute GVHD °II in the MMFD and MUD groups, respectively. One MUD and one MFD recipient developed mild chronic GVHD (skin, oral mucosa). Both are off treatment and without signs of active GVHD at last follow up. Neither acute GVHD ≥ °III nor moderate/severe chronic GVHD was observed in our cohort. There was no significant difference in acute or chronic GVHD incidence between the three donor groups (*p* = 0.086 and 0.421; Table 2; Fig. 2). Immunosuppression was terminated at a median of 135 days (IQR 121–146) after HSCT (Table 2). The disease-free, severe GVHD-free EFS was therefore 80%, 100%, and 100% in the MMFD, MUD, and MFD groups, respectively (*p* = 0.106; Table 2; Fig. 1B).

**Table 2:** Outcome

	All patients		MFD		MUD		MMFD		*p*^a^
*n* =	31		15		6		10		
Median follow-up, months (range)	**26**	**(6–123)**	**26**	**(12–86)**	**29**	**(12–123)**	**14**	**(6–98)**	
Status at last follow-up									
Alive, *n* (%)	31/31	(100%)	15/15	(100%)	6/6	(100%)	10/10	(100%)	*n*/a
Free of SCD, *n* (%)	29/31	(94%)	15/15	(100%)	6/6	(100%)	8/10	(80%)	0.106
Chimerism at last follow-up									
≥ 90% donor	26/31	(84%)	13/15	(87%)	5/6	(83%)	8/10	(80%)	0.482
80–89% donor	2/31	(6%)	1/15	(7%)	1/6	(17%)	0/10	(0%)	
< 80% donor	3/31	(10%)	1/15	(7%)	0/6	(0%)	2/10	(20%)	
Engraftment									
Days post HSCT, median (IQR)									
Neutrophils > 500 × 10/l	20	(17**–**22)	22	(21**–**24)	17	(16**–**19)	18	(16**–**20)	** < 0.001**
Platelets > 50 × 10/l	22	(17**–**25)	22	(18**–**25)	17	(15**–**17)	25	(20**–**32)	0.190
GVHD									
Acute GVHD, *n* (%)									
II	4/31	(13%)	0/15	(0%)	2/6	(33%)	2/10	(20%)	0,086
III–IV°	0/31	(0%)	0/15	(0%)	0/6	(0%)	0/10	(0%)	-
Chronic GVHD, *n* (%)									
Mild	2/31	(6%)	1/15	(7%)	1/6	(17%)	0/10	(0%)	0.421
Moderate/severe	0/31	(0%)	0/15	(0%)	0/6	(0%)	0/10	(0%)	-
End of immunosuppression days post HSCT, median (IQR)	135	(121**–**146)	141	(133**–**143)	146	(122**–**192)	124	(111**–**134)	**0.044**
Acute toxicity									
VOD or TMA, *n* (%)	2/31	(6%)	1/15	(7%)	0/6	(0%)	1/10	(10%)	0.732
Mucositis WHO grade, median (range)	2	(0**–**4)	1	(0**–**3)	2	(1**–**4)	3	(2**–**4)	**0.026**
Organ toxicity ≥ CTCAE °3									
Hepatic (ALT or bilirubine)	5/31	(16%)	0/15	(0%)	0/6	(0%)	5/10	(50%)	**0.002**
Renal (creatinine)	0/31	(0%)	0/15	(0%)	0/6	(0%)	0/10	(0%)	-
Infections									
Viral reactivations, *n* (%)^b^									
Overall	21/31	(68%)	6/15	(40%)	5/6	(83%)	10/10	(100%)	**0.005**
CMV	18/31	(58%)	5/13	(38%)	4/6	(67%)	9/10	(90%)	-
EBV	3/31	(10%)	1/15	(7%)	1/6	(17%)	1/10	(10%)	-
ADV	2/31	(6%)	1/15	(7%)	1/6	(17%)	1/10	(10%)	-
VZV	1/31	(3%)	0/15	(0%)	0/6	(0%)	1/10	(10%)	-
Viral disease, *n* (%)									
Overall	6/31	(19%)	2/15	(13%)	2/6	(33%)	2/10	(20%)	0.576
CMV	1/31	(3%)	1/15	(7%)	0/6	(0%)	0/10	(0%)	-
Other^c^	5/31	(16%)	1/15	(7%)	2/6	(33%)	2/10	(20%)	-
Bacterial BSI	10/31	(32%)	4/15	(27%)	4/6	(67%)	2/10	(20%)	0.125

*ALT* alanine transaminase, *BSI* blood stream infection, *CTCAE* common terminology criteria for adverse events, *IQR* interquartile range, *TMA* thrombotic microangiopathy, *VOD* veno-occlusive disease

^a^Comparison of the three donor groups. Chi square was used for categorical variables, one-way ANOVA for continuous variables

^b^Some patients had more than one virus reactivation and therefore, individual numbers may not add up to overall number

^c^2 × SARS-CoV-2, 1 × BK, 1 × Parvo B19, 1 × Influenza A

### Complications and toxicity

The incidence of veno-occlusive disease (VOD) was overall very low with one MUD and one MMFD patient developing mild VOD (*p* = 0.732; Table 2). There was a significantly higher incidence of acute hepatic toxicity in the MMFD group with 50% of patients experiencing alanine transaminase (but not bilirubine) elevation of CTCAE °3 in the first 100 days (*p* = 0.002). No renal toxicity assessed by serum creatinine elevation was observed (Table 2). Posterior reversible encephalopathy syndrome (PRES) or other CNS toxicities were not observed. Oral mucositis was more severe in the MMFD cohort (median WHO grade 3), compared to the MUD (2) and MFD (1) groups (*p* = 0.026; Table 2). There was a higher incidence of virus reactivation in the MMFD (100%) and MUD (83%) donor groups compared to the MFD group (40%; *p* = 0.005), but not of viral disease (20% vs 33% vs 13%; *p* = 0.576; Table 2). One patient in the MFD group experienced symptomatic CMV disease (fever only, treated successfully with ganciclovir), and one MUD recipient had BK virus–associated hemorrhagic cystitis. No patient developed EBV-associated post transplantation lymphoproliferative disease.

### Engraftment and immune reconstitution

**Fig. 3:**
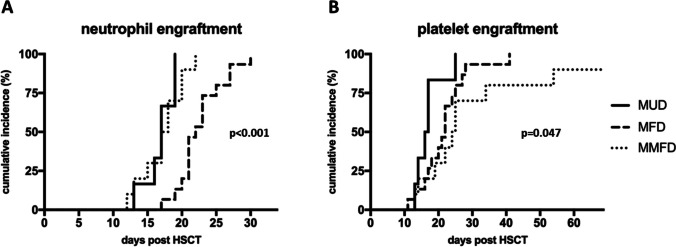
Engraftment. Cumulative incidence of (**A)** neutrophil engraftment (> 0.5 × 10/l) and (**B)** platelet engraftment (> 50 × 10/l) by donor groups

**Fig. 4:**
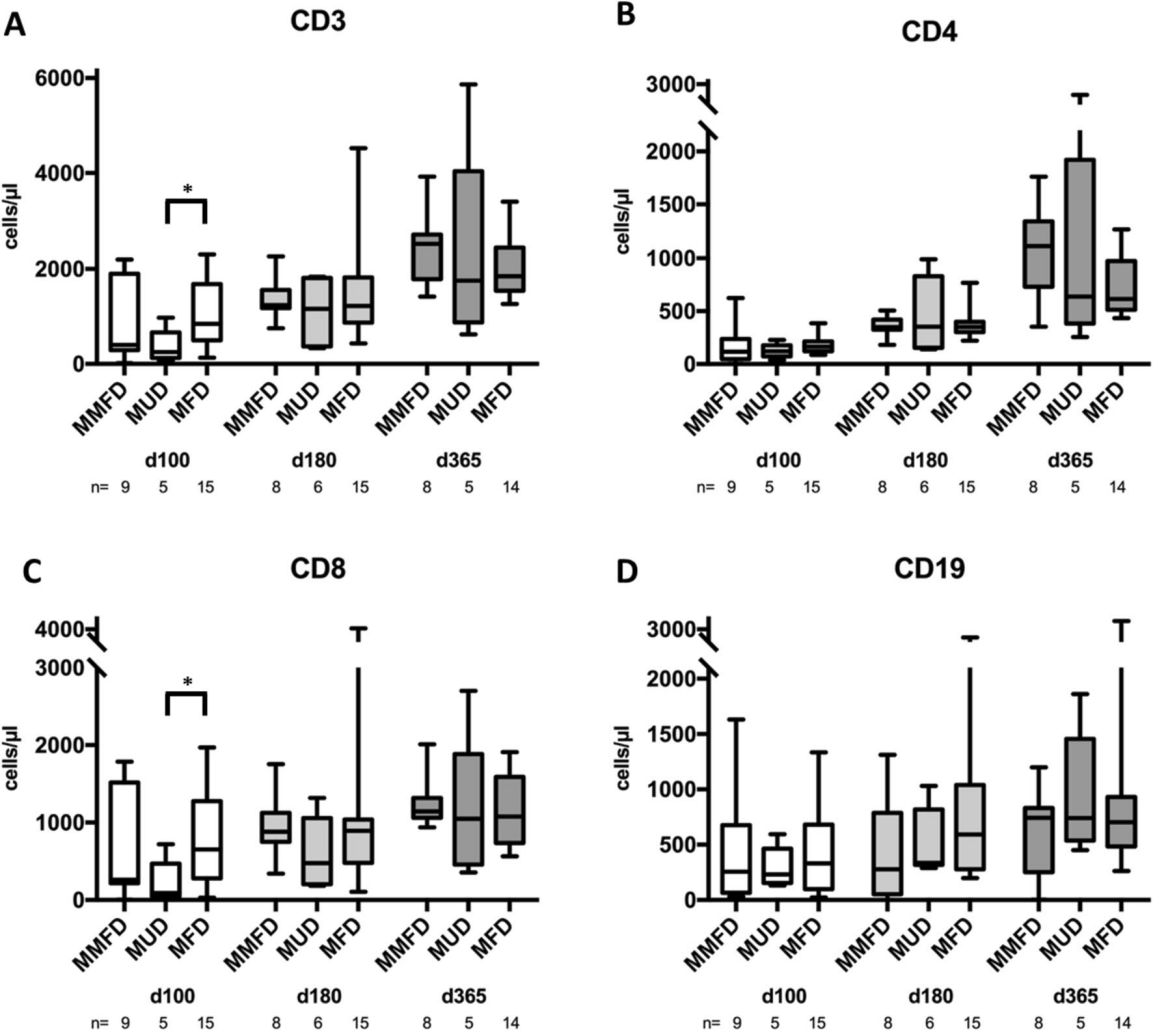
Immune reconstitution. Lymphocyte subsets measured on days 100, 180, and 365 post HSCT by donor groups: **A** CD3 + T-cells, **B** CD4 + T-cells, **C** CD8 + T-cells, and **D** CD19 + B-cells. Statistically significant differences (*p* < 0.05) between two donor groups at a specific time point are indicated by an asterisk (unpaired, two-tailed t-test with Welch correction). Horizontal line represents the mean, boxes the 5th and 95th percentile, and whiskers represent outliers

Neutrophil engraftment occurred significantly later in the MFD group [median (IQR): 18 (16–20) days (MMFD) vs 17 (16–19) days (MUD) vs 22 (21–24) days (MFD), *p* < 0.001], and platelet engraftment occurred slightly later in the MMFD group [median (IQR): 25 (20–32) days (MMFD) vs 17 (15–17) days (MUD) vs 22 (18–25) days (MFD), *p* = 0.047, Table 2; Fig. 3]. The kinetics of cellular immune reconstitution as measured on d + 100, d + 180, and d + 365 was similar across donor groups, with the exception of a slower recovery of CD3 + and CD8 + T-cells at d + 100 in MUD compared to MFD recipients (*p* = 0.013 and *p* = 0.014, respectively; Fig. 4).

### Chimerism and disease status

**Fig. 5:**
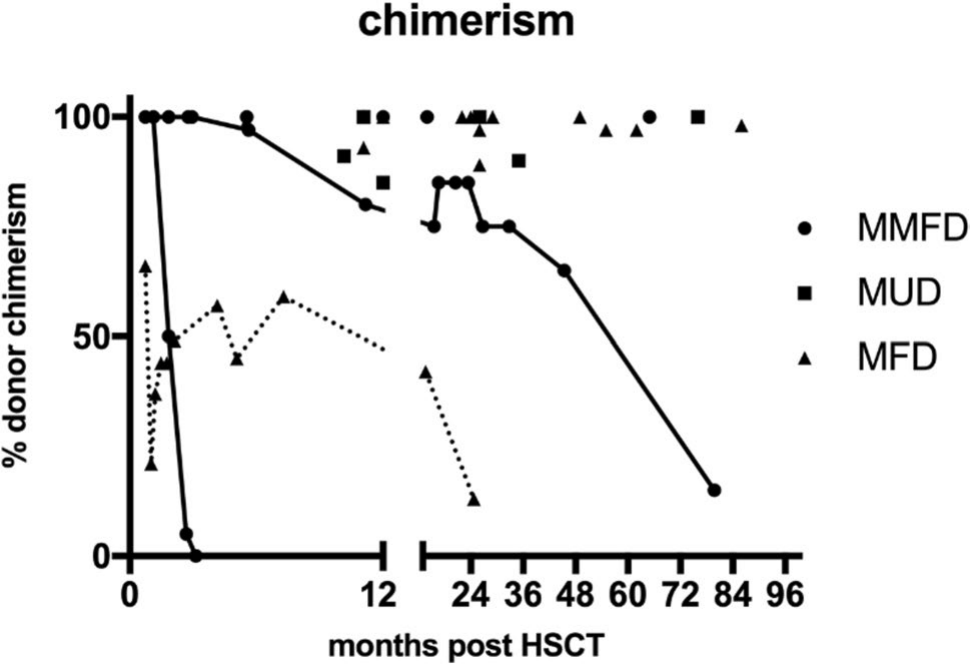
Chimerism. Whole-blood chimerism for all patients by donor groups. Only the last available measurement is shown for patients with ≥ 80% donor chimerism

Whole blood donor chimerism was ≥ 80% in 28/31 patients (90%) at last follow-up (Table 2). Chimerism results at last follow-up and detailed progression of chimerism for the three patients (2 MMFD, 1 MFD) with lower chimerism are shown in Fig. 5. One of the two patients in the MMFD group who experienced secondary graft failure had rapid autologous reconstitution with reappearance of SCD symptoms at 3 months after HSCT, while the other patient had a slow decline until she presented with symptomatic vaso-occlusive crises (VOC) at the 6-year follow-up with 15% donor chimerism and 75% HbS. Both MMFD recipients with graft failure had received treosulfan-based conditioning; neither had an AB0 mismatch. All other patients remain free from SCD-related symptoms, and no VOCs were observed after HSCT. Overall, six patients had a treosulfan-based conditioning, two of whom experienced graft failure (33%), compared to 0/25 (0%) after busulfan-based conditioning (*p* = 0.032).

One MFD recipient, whose sibling donor has sickle cell trait, has 13% whole blood donor chimerism but 36% HbS — which corresponds to the HbS level of his donor — and a normal hemoglobin level of 11.8 g/dl 25 months after HSCT. There was no AB0 blood type mismatch. and there were no minor red blood cell markers available allowing us to demonstrate the presumably complete erythropoetic donor chimerism. All other patients, in whom HbS levels were assessed, either had levels equivalent to their sickle cell trait donors (8/8) or an HbS level of 0% (6/6).

## Discussion

In this retrospective study, we observed 100% overall survival after allogeneic hematopoietic stem cell transplantation (HSCT) for symptomatic sickle cell disease in children, adolescents, and young adults without severe acute or chronic graft-versus-host disease (GVHD), regardless of donor type. Engraftment and immune reconstitution were comparable, and disease-free, severe GVHD-free survival was 100% in MFD and MUD recipients and 80% in MMFD recipients.

While the sample size was too low in this study to make robust comparisons between donor groups, there were some subtle differences in outcome. Two patients with MMFD experienced secondary graft failure with autologous reconstitution. One of them had pre HSCT anti donor HLA antibodies, but the secondary graft failure occurred 6 years after HSCT, making it highly unlikely that these antibodies caused the graft failure. There was no graft failure in any of the other donor groups.

Since SCD is a non-malignant hematologic disorder, avoiding GVHD is of utmost importance. Successful GVHD prevention by in vitro or in vivo lymphodepletion has been reported for haploidentical HSCT in SCD [17–20]. GVHD rates were remarkably low in our patients with the complete absence of severe acute and chronic GVHD in all donor groups including 9/10 MUD and MMFD. We believe this is at least in part due to the use of serotherapy in all patients regardless of donor source, and the young average age of our cohort, which also allowed for early termination of immunosuppression. Other studies have also found that the combination of serotherapy and PTCY is very effective in preventing GVHD in the MMFD setting [16, 29, 30], including a recent study by Oostenbrink et al. who used PTCY with serotherapy for MMFD as well as 9/10 HLA-matched unrelated donors in children with hemoglobinopathies [19]. It has to be noted that the MUD recipients in our cohort did not receive PTCY even though four of the six were only 9/10 matched. Our results are also in line with pooled outcome data recently analyzed in a systematic literature review of predominantly matched donor transplants for SCD [22], as well as with outcomes achieved in pilot trials with in vitro T-cell depletion [18, 31]. Our data suggest that PTCY combined with serotherapy is effective and safe in this donor group [16, 29, 32]. It is important to note that prospective trials in haploidentical HSCT are urgently needed, and one such trial with in vitro T-cell depletion (EudraCT No 2018-002652-33) is currently recruiting. The addition of cyclophosphamide may potentially result in increased long-term toxicity, underlining the necessity for careful long-term follow-up [33, 34].

In the past years, some studies have noted higher rates of mixed chimerism or second HSCT after treosulfan-based conditioning compared to fully myeloablative busulfan in various diseases [35, 36], including a recent retrospective EBMT analysis in thalassemia major [37]. Our conditioning protocol for MMFD and MUD was initially treosulfan-based. However, we observed declining donor chimerism in two of six patients, one of them with anti-donor HLA antibodies pre HSCT. Subsequently, treosulfan was replaced by targeted busulfan with the same target AUC as in MFD recipients after these initial six patients, and no further graft failures were observed. This observation in a limited number of patients does not imply that treosulfan is generally less myeloablative than busulfan, and further exploration — preferably in prospective studies — is required also to define an optimal busulfan target AUC for this population. In general, intensification of myeloablation may be possible in order to avoid graft failure in young SCD patients, but should be explored very carefully in adults [11]. Busulfan has been reported for conditioning in SCD for MSD transplants in younger patients [12], or for adolescents and young adults [38], and is currently being evaluated in the prospective BMT-CTN 1503 trial (ClinicalTrials.gov identifier: NCT02766465). We believe its myeloablation/toxicity ratio can be favorable if used with pharmacokinetic monitoring [39]. The busulfan AUC used in our protocol lies below the reported fully myeloablative AUC, possibly reducing toxicity [39]. However, the addition of thiotepa to the regimen may have helped to ensure durable engraftment. Avoiding graft failure with autologous reconstitution may be especially important in SCD because of the possible risk for secondary hematopoietic neoplasms after graft failure [40, 41].

There was a higher rate of temporary hepatic toxicity (elevated transaminases) and oral mucositis in the MMFD recipients, most likely due to the co-administration of cyclophosphamide in the PTCY protocol [42]. This did not result in an increase in VOD incidence. Overall acute toxicity of the regimen used in this cohort was manageable, underlined by the absence of transplant-related mortality. We observed higher rates of viral reactivations but not viral disease after MMFD and MUD transplantation possibly due to the more potent immunosuppression of alemtuzumab as compared to ATG, and possibly the administration quite proximal to the graft [43]. The rate of viral reactivation was higher in our cohort than in a recent multicenter cohort, possibly explained by different regimens of post HSCT immunosuppression or institutional screening and prophylaxis regimens [44]. The deeper level of immunosuppression with PTCY is known to result in a higher risk of CMV reactivation, which should be carefully monitored and treated pre-emptively after HSCT [44, 45]. There were no stark differences in quantitative T- and B-cell reconstitution between the donor groups, but since this was not a prospective trial, sampling was limited to routine time points starting as late as d + 100, therefore missing potential differences in early T-cell reconstitution. We observed a later recovery of neutrophils in the MFD group, which was also the group with significantly lower total nucleated cell content of the grafts.

HSCT can be transformative for the life of SCD patients, but finding well-matched donors can be challenging. In the absence of a matched donor, MMFD HSCT is a relevant alternative. In vivo manipulation of alloreactive T-cells with PTCY after HSCT from HLA-haploidentical donors may be a good approach for non-malignant diseases like SCD as its use is associated with low GVHD and low graft failure rates in malignant diseases [46]. The published evidence for haploidentical HSCT with PTCY in SCD is still limited, but its application is quickly increasing since the publication of the seminal paper by Bolanos-Meade et al. in 2012 [16, 47]. Reported PTCY case series often comprised pediatric as well as adult patients, included diseases other than SCD, or reported high graft-failure rates [21, 23, 29], but recent data in children are very encouraging [19]. Our data add to the mounting evidence that MMFD HSCT is a viable alternative to matched donor HSCT.

This study has various limitations. Since it was a retrospective, chart-based analysis of clinical data, it is impossible to disentangle the effect of any single confounding factor. The number of patients for each donor type and the follow-up for some patients were limited. Larger prospective studies may shed light on potential pitfalls of our approach, or highlight improved transplant strategies [48]. The excellent success of our reduced toxicity protocols may not be transferrable to other settings, especially not necessarily to older patients with more pre-HSCT organ damage, where less intensive conditioning regimens may be preferred [16, 21, 29]. Nevertheless, our results add to the evidence supporting a more pre-emptive HSCT approach for SCD patients lacking a matched donor [11, 47].

In summary, our institutional protocol with reduced toxicity conditioning resulted in excellent survival and absent severe GVHD. Intensifying myeloablation of conditioning with targeted busulfan may help to ensure durable engraftment.

### Supplementary Information

Below is the link to the electronic supplementary material.

Supplementary file1 (DOCX 13 KB)

## Data Availability

For original, de-identified data, please contact MA at malbert@med.lmu.de.
