# Supplementary material for: Excellent outcome of stem cell transplantation for sickle cell disease
Source: Ann Hematol. 2023 Sep 19;102(11):3217–27. doi: 10.1007/s00277-023-05447-4 (PMC10567813; doi:10.1007/s00277-023-05447-4)
Supplement: Supplementary file 1 — Supplementary file1 (DOCX 13 KB) [file 277_2023_5447_MOESM1_ESM.docx]

**Supplemental table 1: Conditioning regimens**

**MMFD**

| **conditioning drug (daily dose)** | **-9** | **-8** | **-7** | **-6** | **-5** | **-4** | **-3** | **-2** | **-1** | **0** |
| --- | --- | --- | --- | --- | --- | --- | --- | --- | --- | --- |
| alemtuzumab (0,2mg/kg) | • | • |  |  |  |  |  |  |  |  |
| fludarabine (30mg/m^2^) |  |  | • | • | • | • | • |  |  |  |
| busulfan* |  |  | • | • | • |  |  |  |  |  |
| thiotepa (2x5mg/kg) |  | • |  |  |  |  |  |  |  |  |
| cyclophosphamide (14,5mg/kg) |  |  |  |  |  |  | • | • |  |  |

**MUD**

| **conditioning drug (daily dose)** | **-7** | **-6** | **-5** | **-4** | **-3** | **-2** | **-1** | **0** |
| --- | --- | --- | --- | --- | --- | --- | --- | --- |
| alemtuzumab (0,2mg/kg) |  | • | • | • | • |  |  |  |
| fludarabine (30mg/m^2^) |  | • | • | • | • | • |  |  |
| busulfan* |  | • | • | • |  |  |  |  |
| thiotepa (2x5mg/kg) | • |  |  |  |  |  |  |  |

**MFD**

| **conditioning drug (daily dose)** | **-7** | **-6** | **-5** | **-4** | **-3** | **-2** | **-1** | **0** |
| --- | --- | --- | --- | --- | --- | --- | --- | --- |
| ATG Grafalon® (15mg/kg) |  |  |  | • | • | • |  |  |
| fludarabine (30mg/m^2^) |  | • | • | • | • | • |  |  |
| busulfan* |  | • | • | • |  |  |  |  |
| thiotepa (2x5mg/kg) | • |  |  |  |  |  |  |  |

* (weight-based dosing, four times per day, aiming at an AUC of 65-75ng*h/ml)
